# Evaluation of combination protocols of the chemotherapeutic agent FX-9 with azacitidine, dichloroacetic acid, doxorubicin or carboplatin on prostate carcinoma cell lines

**DOI:** 10.1371/journal.pone.0256468

**Published:** 2021-08-25

**Authors:** Franziska Weiner, Jan Torben Schille, Jens Ingo Hein, Xiao-Feng Wu, Matthias Beller, Christian Junghanß, Hugo Murua Escobar, Ingo Nolte

**Affiliations:** 1 Small Animal Clinic, University of Veterinary Medicine Hannover, Hannover, Germany; 2 Department of Medicine, Clinic III, Hematology, Oncology, Palliative Medicine, University of Rostock, Rostock, Germany; 3 Leibniz Institute for Catalysis, Rostock, Germany; Waterford Institute of Technology, IRELAND

## Abstract

The isoquinolinamine FX-9 is a novel potential chemotherapeutic agent showing antiproliferative effects against hematologic and prostate cancer cell lines such as B- and T-acute lymphoblastic leukemia and prostate cancer (PC) of different species. Interestingly, FX-9 shows no hemolytic activity and low toxicity in benign adherent cells. The detailed FX-9 molecular mode of action is currently not fully understood. But application on neoplastic cells induces pro-apoptotic and antimitotic effects. Canine prostate cancer (cPC) represents a unique spontaneous occurring animal model for human androgen-independent PC. Human androgen-independent PC as well as cPC are currently not satisfactorily treatable with chemotherapeutic protocols. Accordingly, the evaluation of novel agent combinations bears significant potential for identifying novel treatment strategies. In this study, we combined FX-9 with the currently approved therapeutic agents doxorubicin, carboplatin, the demethylating substance azacitidine as well as further potentially antitumorigenic agents such as dichloroacetic acid (DCA) in order to evaluate the respective synergistic potential. The combinations with 1–5 μM FX-9 were evaluated regarding the effect after 72 hours on cell viability, cell count and apoptotic/necrotic cells in two human prostate cancer cell lines (LNCaP, PC-3) and a canine prostate cancer cell line (Adcarc1258) representing androgen-dependent and -independent PC/cPC forms. FX-9 in combination with azacitidine decreases cell viability and increases cell death with positive Bliss values. Furthermore, this decreases the cell count with neutral Bliss values on PC-3. Carboplatin in combination with FX-9 reduces cell viability with a neutral Bliss value and increases cell death on LNCaP with calculated positive Bliss values. DCA or doxorubicin in combination with FX-9 do not show synergistic or additive effects on the cell viability. Based on these results, azacitidine or carboplatin in combination with FX-9 offers synergistic/additive efficacy against prostate adenocarcinoma cell lines *in vitro*. The beneficial effects of both combinations are worth further investigation.

## Introduction

FX-9 (3-(p-Tolyl)isoquinolin-1-amine) is a synthesized amino-substituted isoquinoline [1]. Agents of this substance family are antimalarial [2], antifungal [3] and active against different tumors [4–6]. In previous studies, we were able to demonstrate that FX-9 shows an antiproliferative effect on lymphoblastic leukemia cells, inducing morphological changes and apoptosis. Interestingly, cytotoxicity and hemolytic activity against non-neoplastic blood cells were not observed [7]. Furthermore, we reported a pro-apoptotic and anti-mitotic effect of FX-9 on prostate cancer cells of both human and canine origin, with decreased cytotoxic activity in non-malignant chondrocytes and fibroblasts [8].

Therapeutic approaches using compound combinations have been effectively introduced in several treatment protocols in order to increase therapeutic efficacy. Accordingly, combined applications allow resistance mechanisms to be addressed and the enhancement of drug sensitivity against individual agents [9]. Moreover, the agents may act synergistically or additively, allowing lower therapeutic concentrations of the combined agents to be administered, potentially reducing side effects [10]. In this study, FX-9 was combined with four agents with different acting mechanisms. Three of these chemotherapeutic agents were approved by the U.S. Food and Drug Administration (FDA) for the treatment of human cancers: Azacitidine [11], doxorubicin [12] and carboplatin [13]. Azacitidine is used as a DNA-hypomethylating agent in first-line treatment for higher-risk myelodysplastic syndrome (MDS) in humans [14]. A preclinical phase I trial in dogs with urothelial cancer and *in vitro* studies on canine mammary cancer cell lines showed therapeutic potential of this agent [15, 16]. The anthracycline doxorubicin employs many mechanisms of action, of which the basic described mechanism for cell killing is the intercalation into DNA [17]. Therapy with doxorubicin is used for solid tumors and hematological malignancies in both species [18, 19]. Carboplatin, a platinum-based drug, binds to DNA, thereby inhibiting replication and transcription and inducing cell death [20]. It is used in the treatment of solid tumors, for example in humans with ovarian cancer [21] and in dogs with osteosarcomas [22]. Dichloroacetic acid (DCA) inhibits the pyruvate dehydrogenase kinase and has an antiproliferative effect on canine prostate cancer cells [23]. In humans, DCA is not yet in clinical use, but there is a growing body of literature supporting the efficacy of DCA against cancer [24]. Currently, it is not approved by the FDA and not used in the treatment of prostate cancer.

Prostate cancer occurs spontaneously in humans and dogs. A total of 10–20% of human patients develop castration-resistant prostate cancer (CRPC) within five years of diagnosis, and over 84% of CRPC patients have bone metastases [25]. CRPC is currently not treatable, except by surgery or radiation therapy. Taxanes are currently recommended as first option for symptomatic patients with metastatic CRPC [26]. Progression to taxane-resistant prostate cancer, however, occurs in 29% of patients while still receiving docetaxel and in 45% of patients in the first three months after last docetaxel treatment [27]. Dogs showed severe hypersensitivity reactions when treated with the taxane formulations containing the excipient cremophor [28]. Alternative formulations (for example Paccal Vet^®^) were developed to lower hypersensitivity reactions but also side effects such as leucopenia and neutropenia [29]. Paccal Vet^®^ was withdrawn by the pharmaceutical company [30, 31] due to the benefit-risk balance. Combination of FX-9 with taxanes was not performed in the present study as subsequent in vivo research in dogs is currently unlikely due to known side effects of taxanes. The diagnosis in most dogs is a very aggressive late stage metastatic cancer, leading to short-term mortality. CRPC in men and the disease in male dogs are comparable, as they share many similarities, e.g., the embryonic development, the homologous growth, the microscopic anatomy, the occurrence of bone metastases and androgen independency [32]. Therefore, dogs have become a model organism for studying CRPC in humans, and both species could benefit from improved treatment options [32]. In the present study, the well characterized androgen-dependent (LNCaP) and androgen-independent (PC-3) human cell lines, as well as the androgen-independent canine cell line Adcarc1258 were chosen, these being the most common types of prostate cancer in both species.

The aim of the study was to investigate *in vitro* on two human prostate carcinoma cell lines and one canine prostate carcinoma cell line at which FX-9 dosage a combination with azacitidine, DCA, doxorubicin or carboplatin could provide synergistic or additive effects.

## Materials and methods

### Test agents

FX-9 (Leibniz Institute for Catalyses, University of Rostock, Germany) 10 mM stock solution was dissolved in dimethylsulfoxide (DMSO; Merck KGaA, Darmstadt, Germany).

Azacitidine (Absource Diagnostic GmbH, Munich, Germany) 10 mM stock solution was dissolved in DMSO (Merck KGaA).

Dichloroacetic acid (DCA; Merck KGaA, Darmstadt, Germany) was used in ≥ 99% purity and a lot-specific concentration of 1.547 g/mL. A 1 M stock solution was prepared and stored at -4°C for up to four weeks. For the working solution, the DCA stock solution was dissolved in distilled water and adjusted to pH 7 with a sodium hydroxide solution. The final solution was filtered through a 0.22 μm filter.

Doxorubicin (Doxo-Cell 2 mg/mL, STADAPHARM GmbH, Bad Vilbel, Germany) 100 μM stock solution and carboplatin (Carbo-CELL 10 mg/mL, STADAPHARM GmbH) 10 mM stock solution were prepared with culture medium (medium 199 (Gibco^™^, Thermo Fisher Scientific GmbH, Darmstadt, Germany), 10% FBS superior (Biochrom GmbH, Berlin, Germany), 2% penicillin-streptomycin (Biochrom GmbH)) and divided into aliquots.

All test agents were stored at -20°C and different concentrations were prepared directly before each experiment. The FX-9 concentrations 1 μM, 2 μM, 3 μM, 4 μM, 5 μM were in accordance with our preliminary study [8]. The concentrations of the combination partners were chosen to reduce cell viability by approximately 50% (IC50) to show possible effects of the combination with FX-9. In case the respective IC50 exceeded the maximum achievable or tolerable in vivo plasma concentration, the respective maximum concentration was used.

Dihydrotestosterone (DHT; Absource Diagnostic GmbH) was prepared in a 1 mM stock solution by dissolving 5 mg solid DHT in 17.21 mL ethanol absolute (EtOH).

### Cell lines and culture

TiHoDProAdcarc1258 (Adcarc1258) is a canine cell line, derived from a prostate adenocarcinoma of a ten-year-old male Briard [33, 34]. LNCaP is an androgen-sensitive human cell line of a prostate carcinoma metastasis. The cells originate from the left supraclavicular lymph node of a 50-year-old Caucasian man [35]. PC-3 is an androgen-insensitive human cell line derived from bone metastasis from a prostate carcinoma of a 62-year-old man [36]. The cell lines were cultivated in 25 cm^2^ cell culture flasks in medium 199 (Gibco^™^) with 10% FBS superior (Biochrom GmbH) and 2% penicillin-streptomycin (Biochrom GmbH). The cell cultures were cultivated at 37°C and 5% CO_2_ in a humidified atmosphere.

### Cell viability

Metabolic activity was measured using the CellTiter 96^®^ AQueous One Solution Cell Proliferation Assay (Promega GmbH, Walldorf, Germany). Cells were seeded in 96 well plates at a density of 7,500 cells per well and allowed to adhere overnight. Cells were exposed to 1 μM, 2 μM, 3 μM, 4 μM and 5 μM FX-9 single application and in combination with 3 μM azacitidine, 3 mM DCA, as well as cell line specific dosages of 75 nM (Adcarc1258)/100 nM doxorubicin (PC-3 and LNCaP) or 20 μM (Adcarc1258)/60 μM (PC-3)/80 μM (LNCaP) carboplatin. Single applications of Azacitidine, DCA, doxorubicin and carboplatin were tested at the concentrations outlined above. After 72 hours, 100 μL of MTS (3-(4,5-dimethylthiazol-2-yl)-5-(3-carboxymethoxyphenyl)-2-(4-sulfophenyl)-2H-tetrazolium, inner salt) dissolved in culture medium were added to each well. After two hours of incubation, the absorbance at 490 nm was measured with the Multi-Mode Reader Synergy 2 (BioTek Instruments GmbH, Bad Friedrichshall, Germany). The amount of formazan produced by the cells by reducing MTS is proportional to the viability of the cells. The mean value of four wells was used per concentration and per experiment. The experiment was performed in triplicates.

### Bliss independence model

Synergistic effects of drug combinations were calculated using the Bliss independence model. This mathematical method is used to compare the predicted effect (E_P_) with the observed effect (E_O_). E_P_ is calculated according to the formula: E_P_ = E_a_ + E_b_—E_a_E_b_. Ea and Eb represent the effects of the single application of the agents. The difference between the predicted and observed effect is the Bliss value, which determines the degree of synergy. Bliss values greater than zero indicate a synergistic effect, equal to zero an additive effect and less than zero an antagonistic effect [37]. Synergistic or additive effects were confirmed by a significant higher efficacy of the combined agents compared to the single application of FX-9 and of azacitidine, DCA, doxorubicin or carboplatin.

### Cell count

The three cell lines were seeded in 6-well plates at a density of 100,000 cells per well in culture medium and allowed to adhere overnight. The cells were exposed to 1 μM, 2 μM and 3 μM FX-9 single application and in combination with 3 μM azacitidine or 20 μM (Adcarc1258)/80 μM (LNCaP) carboplatin. Single applications of azacitidine and carboplatin were tested in aforementioned concentrations. The cells were incubated at 37°C and 5% CO_2_ in a humidified atmosphere. After 72 hours, the cells were harvested with TrypLE^™^ Express Enzyme (Gibco^™^, Thermo Fisher Scientific GmbH) and counted with an automatic cell counter (Cellometer^™^ Auto T4, Nexcelom Bioscience LLC, Lawrence, MA, USA). The mean value of three wells was used per concentration and per experiment. The experiment was performed in biological triplicate.

### Analysis of apoptosis

The apoptosis rate was analyzed by flow cytometry in all three cell lines after 72 hours of compound exposure. Adherent cells and culture medium were collected to analyze the non-attached cells as well. Samples were pelleted and resuspended in 250 μL binding buffer (Annexin V-FITC Detection Kit plus, PromoCell GmbH, Heidelberg, Germany) before being filtered through a 70 μm filter. Subsequently, 2.5 μL Annexin V-FITC (PromoCell GmbH) and 0.5 μL TO-PRO-3 iodide (Thermo Fisher Scientific Inc., Waltham, MA, USA) were pipetted onto each sample. MACSQuant^®^ Analyzer 10 (Miltenyi Biotec B.V. & Co. KG, Bergisch Gladbach, Germany) was used for the flow cytometric measurements. Data analysis was performed with the software FlowJo 7.6.5 (FlowJo, LLC, Ashland, OR, USA). The mean value of three wells was used per concentration and per experiment. The experiment was performed in biological triplicate.

### Androgen sensitivity

The cell lines Adcarc1258, PC-3 and LNCaP were seeded in 6-well plates at different seeding densities (25,000 cells (Adcarc1258)/15,000 cells (PC-3)/100,000 cells (LNCaP) per well) due to different growth behavior and exclusion of growth inhibition by cell contacts. Cells adhered over night and were exposed to 10 nM DHT [35] or with an equivalent volume of EtOH in culture medium over a 120-hours period in a humidified atmosphere with 37°C and 5% CO_2_. After incubation, the cells were harvested by TrypLE^™^ Express Enzyme (Gibco^™^, ThermoFisher Scientific GmbH) and counted with an automatic cell counter (Cellometer^™^ Auto T4, Nexcelom Bioscience LLC). The mean value of three wells per concentration and per experiment was used. The experiment was performed in biological quadruplicate.

### Statistics

Statistical analysis was performed with SAS Enterprise Guide 7.1 (SAS Institute Inc., Cary, NC, USA). The values of the measurements were tested for normal distribution. The student’s t-test was used for LNCaP and Adcarc1258, and the Wilcoxon test for PC-3 to calculate p-values in the androgen sensitivity experiment. Dunnett’s t-test was used to calculate p-values in the remaining experiments. p < 0.05 was considered statistically significant.

## Results

### Combination of FX-9 with azacitidine or carboplatin provides higher efficacy in cell viability

**Fig 1:**
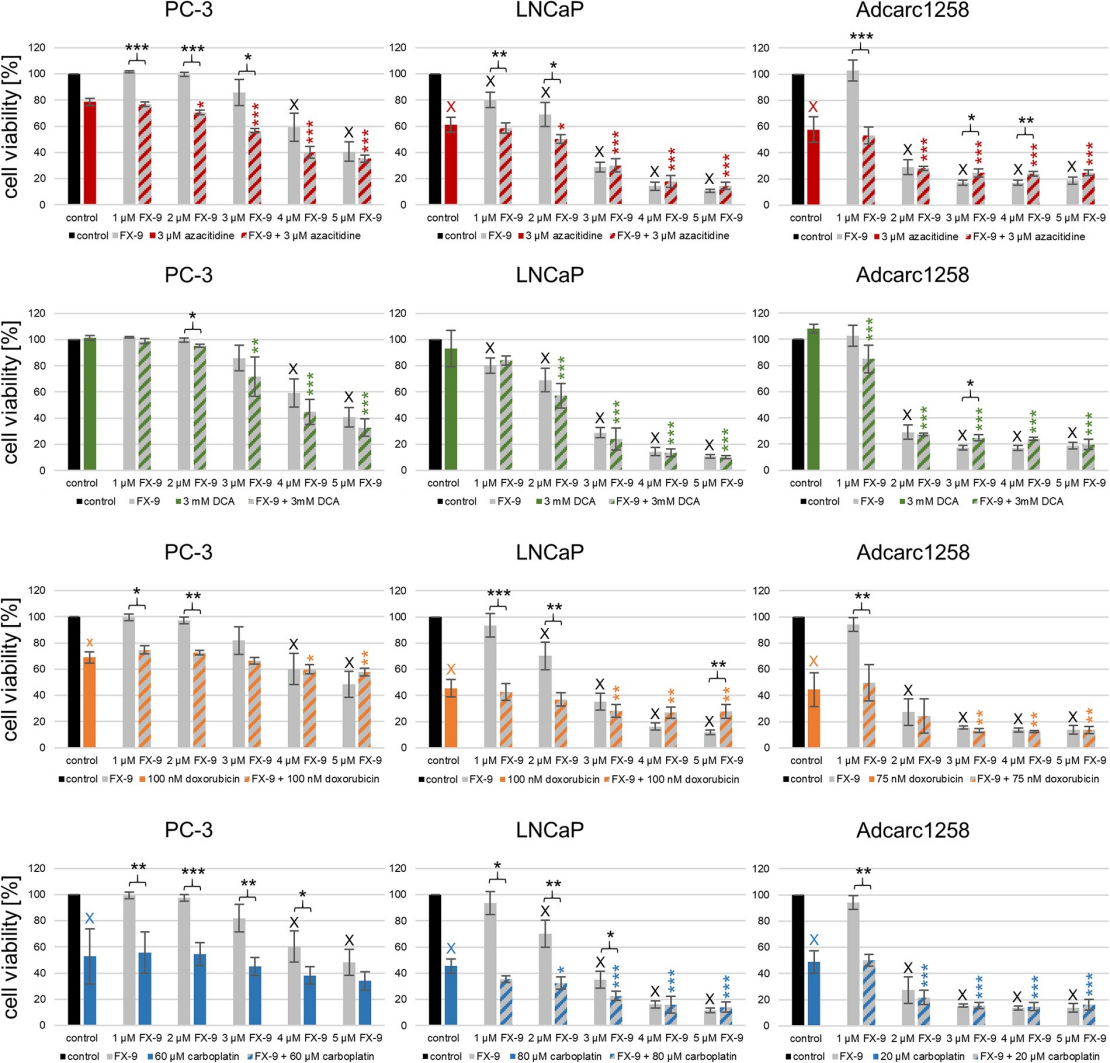
Cell viability of PC-3, LNCaP and Adcarc1258 after single application of FX-9, azacitidine, DCA, doxorubicin or carboplatin and the combinations. The results are expressed as a percentage of DMSO-controls and are plotted as mean ± standard deviation (SD) of three independent experiments. Significance of an effect compared to the single application of FX-9, azacitidine, DCA, doxorubicin, carboplatin or control was calculated by the Dunnett’s t-test. *:p<0.05, **:p<0.01, ***:p<0.001, black *: Significant difference between combination and FX-9 single application, colored *: Significant difference between combination and azacitidine, DCA, doxorubicin or carboplatin single application; x:p<0.01, X:p<0.001, black X: significant difference between single application of FX-9 and DMSO-control, colored X: Significant difference between single application of azacitidine, DCA, doxorubicin or carboplatin and DMSO-control.

A dose-dependent decrease in viability was observed in all cell lines by FX-9 single application starting at 1 μM/2 μM (LNCaP, Adcarc1258) and 4 μM (PC-3). The combinations with azacitidine, DCA, doxorubicin and carboplatin resulted in a dose-dependent and cell line-specific significant decrease in cell viability compared to the single application of the two combined agents (Fig 1).

In detail, the combination of all tested FX-9 concentrations with azacitidine decreased cell viability significant to the respective DMSO-controls in PC-3, LNCaP and Adcarc1258 (S1 Table). Compared to the single applications of FX-9, 1–3 μM (PC-3)/1-2 μM (LNCaP)/1 μM (Adcarc1258) FX-9 with azacitidine resulted in a reduction in cell viability. A reduction in cell viability was shown in all three cell lines starting 2 μM FX-9 compared to azacitidine single application.

All tested FX-9 concentrations with DCA reduced cell viability in LNCaP and Adcarc1258 compared to the DMSO control, whereas in PC-3, the reduction started at 3 μM FX-9. The combination with DCA decreased the cell viability significantly only with 2 μM FX-9 compared to FX-9 single application in PC-3. FX-9 combinations 3–5 μM (PC-3)/2-5 μM (LNCaP)/1-5 μM (Adcarc1258) with DCA resulted in a reduction in cell viability compared to the single application of DCA.

The combination of all tested FX-9 concentrations with doxorubicin decreased the cell viability to the respective DMSO-controls in PC-3, LNCaP and Adcarc1258 significantly. Doxorubicin with 1–2 μM (PC-3, LNCaP)/1 μM (Adcarc1258) FX-9 caused a lower cell viability than FX-9 single applications. FX-9 combinations starting at 4 μM (PC-3) or 3 μM (LNCaP, Adcarc1258) resulted in reduced cell viabilities compared to a single application of doxorubicin.

The combination of all tested FX-9 concentrations with carboplatin decreased cell viability significant to the respective DMSO-controls in PC-3, LNCaP and Adcarc1258. Carboplatin with 1–4 μM (PC-3)/1-3 μM (LNCaP)/1 μM (Adcarc1258) FX-9 decreased cell viability compared to single application of FX-9. In comparison with the single application of carboplatin, no combination with FX-9 in PC-3 and combination with 2–5 μM FX-9 in LNCaP and Adcarc1258 caused a reduction in cell viability.

### Synergistic interactions for FX-9 with azacitidine, additive effects of FX-9 and carboplatin

Bliss values for the combination of FX-9 with azacitidine gave positive values for 1 μM FX-9 on Adcarc1258 and 2 μM, 3 μM, 4 μM FX-9 on PC-3 (Table 1). For 1 μM and 5 μM FX-9 combined with azacitidine on PC-3, neutral Bliss values were calculated. Bliss values for the combination of 2–5 μM FX-9 with DCA were positive on PC-3. The combination of 2 μm FX-9 (LNCaP) or 1 μM FX-9 (Adcarc1258) with DCA also resulted in positive Bliss values. Neutral Bliss values were calculated for the combination of 1 μM FX-9 (PC-3), 3–4 μM FX-9 (LNCaP), 2 μM and 5 μM FX-9 (Adcarc1258) with DCA. The combination of 1 μM or 2 μM FX-9 with doxorubicin resulted in neutral Bliss values in LNCaP. The combination of 1 μM FX-9 with carboplatin gave a positive Bliss value. Neutral Bliss values were calculated for the combinations of 1–3 μM (PC-3)/2 μM (LNCaP)/1 μM (Adcarc1258) FX-9 with carboplatin. The Bliss values of the combinations of FX-9 with the different substances, which were not previously mentioned in detail, were negative.

**Table 1:** Bliss evaluation of the results of cell viability of FX-9 combination with azacitidine, DCA, doxorubicin or carboplatin in PC-3, LNCaP and Adcarc1258.

*3 μM azacitidine +*	*PC-3*			*LNCaP*			*Adcarc1258*		
	A	B	Bliss value	A	B	Bliss value	A	B	Bliss value
*1 μM FX-9*	***	-	0	**	-	-0.1	***	-	0.1
*2 μM FX-9*	***	*	0.1	*	*	-0.1	-	***	-0.1
*3 μM FX-9*	*	***	0.1	-	***	-0.1	-	***	-0.1
*4 μM FX-9*	-	***	0.1	-	***	-0.1	-	***	-0.1
*5 μM FX-9*	-	***	0	-	***	-0.1	-	***	-0.1
*3 mM DCA +*	PC-3			LNCaP			Adcarc1258		
	A	B	Bliss value	A	B	Bliss value	A	B	Bliss value
*1 μM FX-9*	-	-	0	-	-	-0.1	-	***	0.3
*2 μM FX-9*	*	-	0.1	-	***	0.1	-	***	0
*3 μM FX-9*	-	**	0.2	-	***	0	-	***	-0.1
*4 μM FX-9*	-	***	0.2	-	***	0	-	***	-0.1
*5 μM FX-9*	-	***	0.1	-	***	0	-	***	0
*100/75 nM doxorubicin +*	PC-3			LNCaP			Adcarc1258		
	A	B	Bliss value	A	B	Bliss value	A	B	Bliss value
*1 μM FX-9*	*	-	-0.1	***	-	0	**	-	-0.1
*2 μM FX-9*	**	-	-0.1	**	-	0	-	-	-0.1
*3 μM FX-9*	-	-	-0.1	-	**	-0.1	-	**	-0.1
*4 μM FX-9*	-	*	-0.2	-	**	-0.2	-	**	-0.1
*5 μM FX-9*	-	**	-0.2	-	**	-0.2	-	**	-0.1
*60/80/20 μM carboplatin +*	PC-3			LNCaP			Adcarc1258		
	A	B	Bliss value	A	B	Bliss value	A	B	Bliss value
*1 μM FX-9*	**	-	0	***	-	0.1	**	-	0
*2 μM FX-9*	***	-	0	**	*	0	-	***	-0.1
*3 μM FX-9*	**	-	0	*	***	-0.1	-	***	-0.1
*4 μM FX-9*	*	-	-0.1	-	***	-0.1	-	***	-0.1
*5 μM FX-9*	-	-	-0.1	-	***	-0.1	-	***	-0.1

A: higher efficacy compared to the single application of FX-9, B: higher efficacy to the single application of azacitidine, DCA, doxorubicin or carboplatin. Bliss values >0 represent a synergistic effect, Bliss values = 0 represent an additive effect and Bliss values <0 represent an antagonistic effect. Synergism or additivity requires a higher efficacy compared to the combined two agents single application and a positive or neutral Bliss value. Significance of an effect compared to the single application of FX-9, azacitidine, DCA, doxorubicin or carboplatin was calculated by the Dunnett’s t-test.

*:p<0.05,

**:p<0.01,

***:p<0.001,

-:no significance.

### FX-9 with azacitidine decreases cell count in PC-3

**Fig 2:**
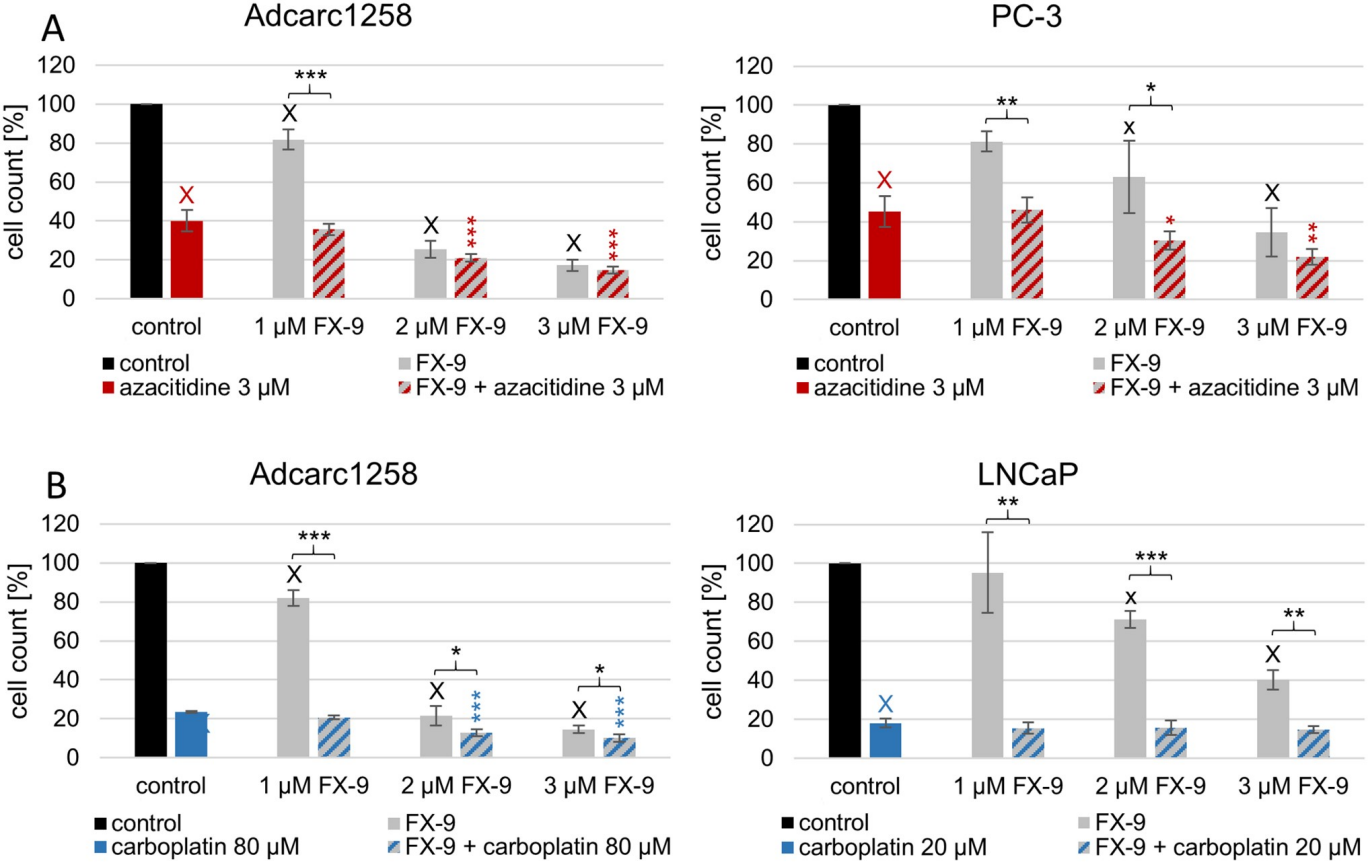
Cell count of PC-3, Adcarc1258 and LNCaP after application of FX-9, A: Azacitidine and B: Carboplatin and both agents in combination. The results are expressed as a percentage of DMSO-controls and are plotted as mean ± standard deviation (SD) of three independent experiments. The significance of an effect compared to the single application of FX-9, azacitidine, carboplatin or DMSO-control calculated by Dunnett’s t-test: *:p<0.05, **:p<0.01, ***:p<0.001, x:p<0.01, X:p<0.001.

The concentrations of FX-9 with azacitidine or carboplatin that significantly decreased cell viability with positive or neutral Bliss values in the human cell lines (PC-3, LNCaP) were further tested using cell count. As an interspecific comparison, the canine cell line Adcarc1258 was also tested. A dose-dependent reduction in cell count after single application of FX-9 was observed starting at 1 μM FX-9 in Adcarc1258, and at 2 μM FX-9 in PC-3 and LNCaP (Fig 2). The combination with azacitidine or carboplatin decreased the cell count significantly compared to the DMSO-control in all tested concentrations and cell lines (S2 Table). Compared to the single application of FX-9, the combination of azacitidine with 1 μM FX-9 reduced the cell count in Adcarc1258 (p<0.001) and PC-3 (p<0.01). The combination with 2 μM FX-9 reduced the cell count to 30.4% in PC-3 (p<0.05) from 63% after single application of FX-9. The combination with 2 μM or 3 μM FX-9 resulted in a lower cell count in both tested cell lines compared to the single application of azacitidine. The cell count was reduced by combining carboplatin and FX-9 in all tested combinations on Adcarc1258 and LNCaP compared to the single application of FX-9. Carboplatin in combination with 2 μM and 3 μM FX-9 reduced the cell count compared to the single application of carboplatin on Adcarc1258 (p<0.001). For LNCaP, no combination was able to reduce the cell count compared to the 18.8% cell count of single application of carboplatin. Azacitidine and FX-9 in combination resulted in the majority of the tested concentrations in negative Bliss values (S3 Table). Exceptions were the neutral values caused by 1 μM FX-9 (Adcarc1258) and 2 μM FX-9 (PC-3) in combination with 3 μM azacitidine. Carboplatin combined with 1 μM FX-9 resulted in neutral Bliss values on both tested cell lines. The combination with 2 μM FX-9 caused a negative value in Adcarc1258 and a neutral value in LNCaP. Negative Bliss values were calculated for the 3 μM FX-9 combination with carboplatin on both cell lines.

### FX-9 induces apoptosis with azacitidine on PC-3 and carboplatin on LNCaP

**Fig 3:**
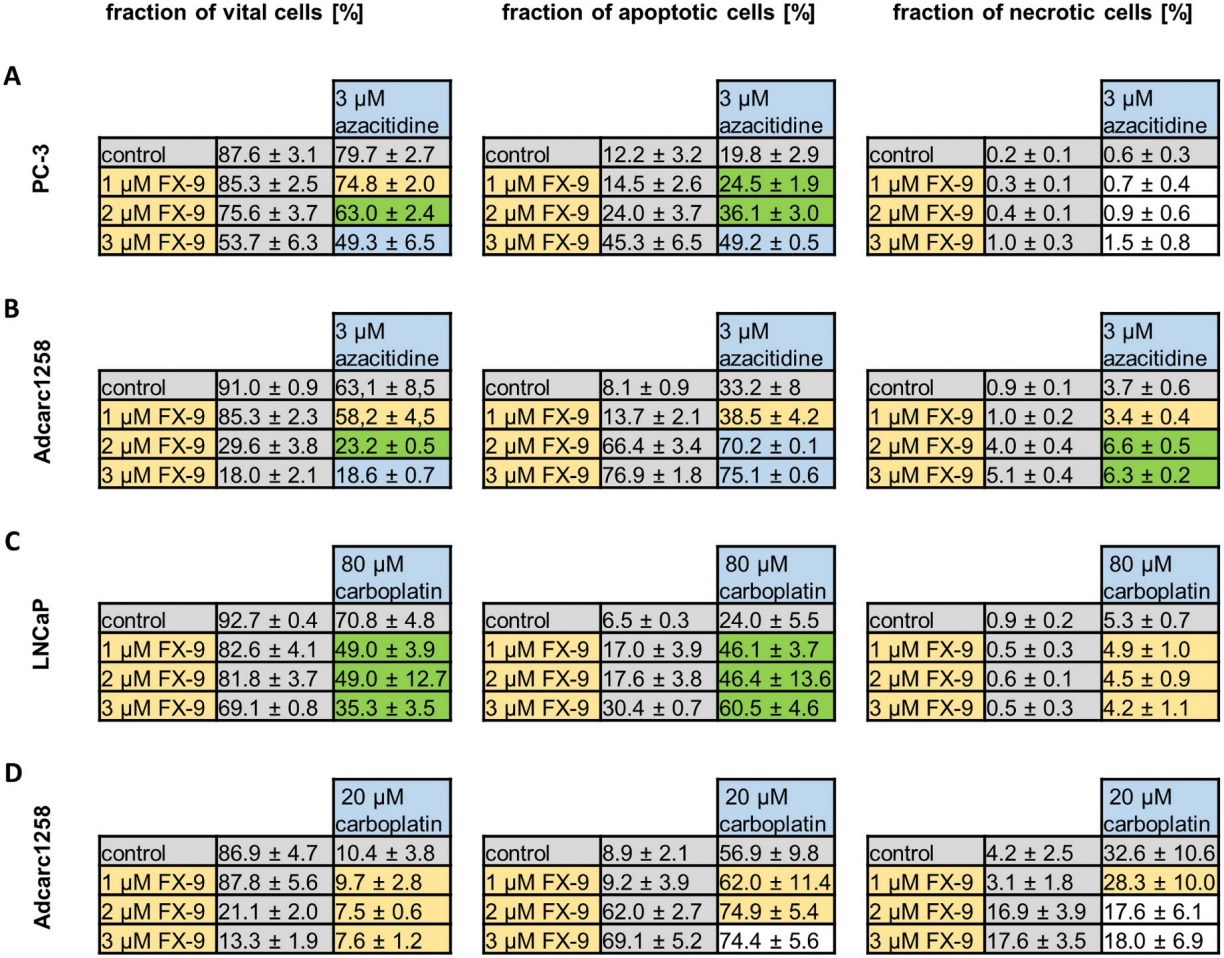
Analysis of apoptosis. **A:** PC-3 after application of FX-9 and azacitidine, **B:** Adcarc1258 after application of FX-9 and azacitidine, **C:** LNCaP after application of FX-9 and carboplatin and **D:** Adcarc1258 after application of FX-9 and carboplatin. The analysis was carried out via flow cytometry with an annexin and TO-PRO-3 iodide staining. Fractions of necrotic, apoptotic and vital cells served as a percentage of the total amount of cells. The results are plotted as mean ± standard deviation (SD) of three independent experiments. Significance of an effect compared to the single application of FX-9, azacitidine or carboplatin was calculated by the Dunnett’s t-test. Color coding: White: No significant difference to both single applications; yellow: p<0.05 compared to FX-9 single application; blue: p<0.05 compared to azacitidine or carboplatin single application; green: p<0.05 compared to both single applications.

The concentrations of FX-9 with azacitidine or carboplatin that significantly decreased cell viability with positive or neutral Bliss values in the human cell lines (PC-3, LNCaP) were further tested using flow cytometry with annexin and TO-PRO-3 iodide staining. As an interspecific comparison, the canine cell line Adcarc1258 was also tested. FX-9 in combination with azacitidine or carboplatin decreased the fraction of vital cells, and increased the fraction of apoptotic cells in all tested cell lines and concentrations significantly compared to the DMSO-control (S4 Table). The fraction of necrotic cells was significant increased except for the combinations 1 μM/2 μM FX-9 with azacitidine in PC-3, and the combinations 2 μM/3 μM FX-9 with carboplatin in Adcarc1258. In the cell line PC-3, the combination of 2 μM FX-9 with azacitidine caused a reduction to 63.0% vital cells compared to the effect of single applications of FX-9 (75.6%) and azacitidine (79.7%) (Fig 3). The calculated Bliss value was neutral (S5 Table). The fraction of apoptotic cells was increased to 24.5% (1 μM FX-9) or 36.1% (2 μM FX-9) by the combination with azacitidine (p:<0.05) with a positive Bliss value. In Adcarc1258, the combination of 2 μM FX-9 with azacitidine reduced the fraction of vital cells compared to the single applications of both agents (p:<0.05), with a calculated neutral Bliss value. The fraction of necrotic cells was increased to 6.6% by combining 2 μM FX-9 with azacitidine compared to 4.0% with a single application of FX-9 and 3.7% with a single application of azacitidine. The combination 3 μM FX-9 with azacitidine also increased the necrotic cells compared to the single applications of FX-9 and azacitidine. A neutral Bliss value was calculated for the effect of this concentration (S2 Table). In LNCaP, all concentrations of FX-9 in combination with carboplatin resulted in a reduction in the proportion of viable cells, and an increase in the proportion of apoptotic cells by about 30 percentage points compared to the single application of FX-9 (p:<0.05). This decrease in vital cells and increase in apoptotic cells were also significant compared to the effect on the cell fractions of an azacitidine single application. Bliss values in all tested FX-9 concentrations with carboplatin were positive for both vital and apoptotic cells on LNCaP. For Adcarc1258, no combination produced a beneficial effect for either single application.

### Adcarc1258 is an androgen-independent cell line

**Fig 4:**
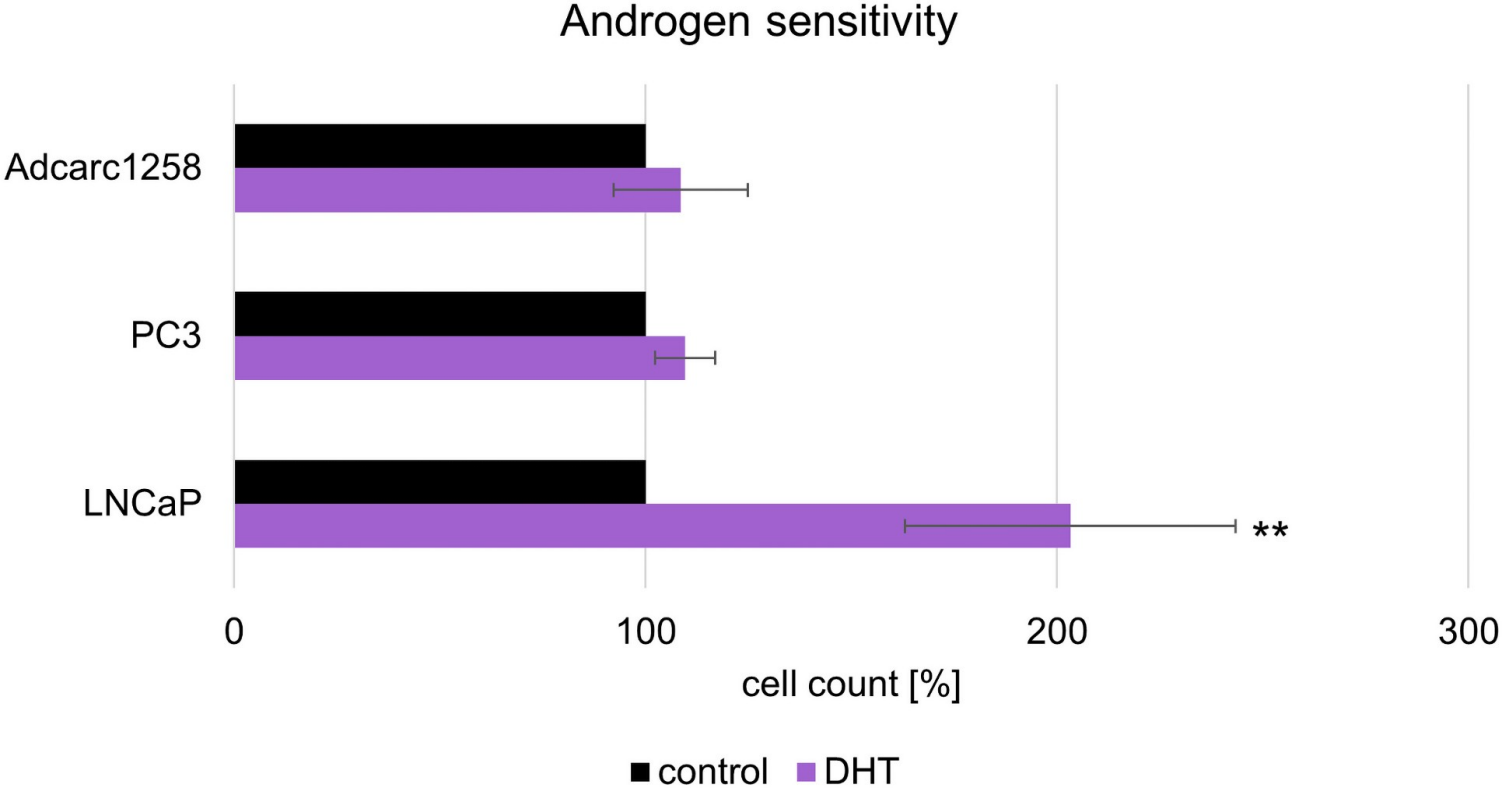
Androgen sensitivity. Cell count of the cell lines PC-3, Adcarc1258 and LNCaP after exposure to DHT for 120 hours. The results are expressed as percentage of EtOH-controls and are plotted as mean ± standard deviation (SD) of three independent experiments. Significance of an effect compared to the EtOH-control was calculated by the students t-test: *:p<0.05.

In the human cell line PC-3 and the canine cell line Adcarc1258 there was no significant difference in cell growth between the control and presence of DHT (Fig 4). With LNCaP, however, the cell count in the presence of DHT was twice as high as in the control.

## Discussion

The aim of this study was to evaluate at which FX-9 dosage a combination with azacitidine, DCA, doxorubicin or carboplatin could provide synergistic or additive effects on human and canine prostate cancer cell lines. Azacitidine, DCA, doxorubicin or carboplatin were tested in concentrations achievable in plasma [38–41] and showed antiproliferative effects in combination with FX-9 on PC and cPC cell lines. Similar to previous results [8], FX-9 solely leads to a dose-dependent reduction in cell viability and cell count in all cell lines. In comparison to this, two of the four tested agents, azacitidine and carboplatin, led to additive or synergistic effects on cell viability in different ways. However, the other two tested agents, DCA and doxorubicin, showed no synergism or additivity. Combination protocols of FX-9 with taxanes were not performed in the present study despite their relevance in human medicine as therapeutic use of taxanes induced severe side effects in dogs [28, 29]. In human medicine, a combination of FX-9 with taxanes remains an interesting option to be further evaluated in additional studies.

Azacitidine is an FDA-approved [11] cytidine analog, and its antineoplastic effects are caused, for example, by hypomethylation of DNA [42] and induction of DNA damage [43]. Azacitidine showed positive effects in a combination therapy, e.g. on platinum-resistant ovarian cancer cells [44] and against myelodysplastic syndrome [45]. In the present study, the combination of azacitidine with the anti-mitotic agent FX-9 showed similar synergistic effects on cell viability on the cell line PC-3. Cell count analysis showed only an additive effect of this combination on cell count on PC-3. Again, synergism for FX-9 with azacitidine on PC-3 was confirmed by analysis of apoptosis. In summary, this combination showed a synergistic effect on apoptosis and an additive effect on vital cells after exposure to 2 μM FX-9. In contrast to this, no synergistic or additive effect was demonstrated on cell viability or cell count in the androgen-independent canine prostate cancer cell line Adcarc1258. However, there was a synergistic effect on increasing the fraction of necrotic cells, and an additive effect on decreasing the fraction of vital cells.

Carboplatin, which was approved by the FDA in 1989 [13], is used to treat many types of cancer [46]. It induces cell death by efficiently binding to DNA and inhibiting replication and transcription [20]. In our study, 80 μM carboplatin in combination with the anti-mitotic drug FX-9 showed an additive effect on cell viability on the cell line LNCaP. However, the cell count was not reduced compared to the single application of carboplatin. On the other hand, the fraction of apoptotic cells increased and vital cells decreased, both with a synergistic effect by exposure to all tested combinations of FX-9 with carboplatin on LNCaP cells. The beneficial effect of the combination of an antimitotic agent and carboplatin is used, for example, in the treatment of patients with advanced non-small cell lung cancer [47]. In contrast to this improved efficacy, the combination with carboplatin did not synergistically or additively increase the efficacy of FX-9 on the cPC cell line, either in cell viability and cell number or in the analysis of apoptosis.

When comparing the cell viability and cell counting methods, differences are evident. Looking at the results of the cell count assay, the compounds appear to be significantly more effective at many concentrations than the cell viability results suggest. This effect was distinct in PC-3, as the combination of FX-9 with azacitidine showed 20–50 percentage points higher efficacy in the cell number compared to cell viability. This phenomenon is well known for the response of cancer cells to DNA synthesis-targeting agents [48]. Responsible for this was a dose-dependent change in the cell phenotype, which was an enlargement in individual cells and consequently mitochondrial content. These cells exhibited a higher MTS-reducing activity per cell, which led to a calculated higher cell viability, although the cell number decreased [48]. FX-9 causes morphological changes, such as enlargement and polyploidy of cells [8]. This could be responsible for the different assessment efficacy in the MTS assay.

The measured synergism was selective regarding concentration and cell line. Synergistic or additive effects occurred dose-dependent at low concentrations of FX-9. It is possible that FX-9 exhibits a dose-dependent mechanism of action, leading to synergism or additivity with azacitidine or carboplatin at low concentrations. There is evidence that antimitotic agents cause drug- and concentration-dependent variation in cell response [49]. Paclitaxel, for example, causes multipolar divisions at lower concentrations and cell killing by a robust mitotic arrest at higher concentrations [50]. The selectivity regarding cell lines, despite the human origin of LNCaP and PC-3, may be due to the differences in expression of the androgen receptor and p53. LNCaP expresses the androgen receptor and the wild-type functional p53, while PC-3 does not express either [51–53]. Additionally, the cell lines express a pattern of unique genes, which represents the aggressive phenotype in PC-3, in contrast to many prostate cell-specific characteristics in LNCaP [54]. The interspecific differences in synergism and additivity of the tested combinations, could be caused by the higher efficiency of FX-9 starting from 2 μM on the canine cell line.

Prostate cancer in humans is usually dependent on androgen and is treated by hormone deprivation therapy. CRPC develops from this disease despite therapy [25]. The two human cell lines, LNCaP and PC-3, are well described and represent human androgen-dependent (LNCaP) and androgen-independent (PC-3) prostate cancer in this study [35, 36]. The canine cell line, Adcarc1258, has been characterised [33, 34], but not previously tested for androgen sensitivity. In the present study, androgen independency was detected. Therefore, this cell line is appropriate and representative for canine prostate carcinomas, as the majority of these are androgen-independent and do not respond to hormonal therapy [32].

In conclusion, FX-9 provides a dose-dependent selective synergistic potential with azacitidine and carboplatin. The cause of this selective potential should be further investigated regarding possible dose-dependent mechanisms of action. The variance between cell lines after exposure to FX-9 supports the tumour-specific use of chemotherapeutic agents for optimal treatment of prostate cancer [55]. Further studies on tolerability, pharmacokinetics and possible routes of drug administration are needed.

## Supporting information

S1 Fig Representative dot blots of flow cytometry data. PC-3 was stained with Annexin V-FITC and TO-PRO-3 iodide after exposure to FX-9 and azacitidine. Cells in area Q4 were counted as vital, cells in Q3 as apoptotic, and cells in Q1 and Q2 as necrotic.
(TIF)

S2 Fig Representative dot blots of flow cytometry data. Adcarc1258 was stained with Annexin V-FITC and TO-PRO-3 iodide after exposure to FX-9 and azacitidine. Cells in area Q4 were counted as vital, cells in Q3 as apoptotic, and cells in Q1 and Q2 as necrotic.
(TIF)

S3 Fig Representative dot blots of flow cytometry data. LNCaP was stained with Annexin V-FITC and TO-PRO-3 iodide after exposure to FX-9 and carboplatin. Cells in area Q4 were counted as vital, cells in Q3 as apoptotic, and cells in Q1 and Q2 as necrotic.
(TIF)

S4 Fig Representative dot blots of flow cytometry data. Adcarc1258 was stained with Annexin V-FITC and TO-PRO-3 iodide after exposure to FX-9 and carboplatin. Cells in area Q4 were counted as vital, cells in Q3 as apoptotic, and cells in Q1 and Q2 as necrotic.
(TIF)

S1 Table Effect of combinations on cell viability. (DOCX)

S2 Table Effect of combinations on cell count. (DOCX)

S3 Table Bliss values of the cell count. (DOCX)

S4 Table Effect of combinations on cell fractions. (DOCX)

S5 Table Bliss values of the analysis of apoptosis. (DOCX)
