# Supplementary figures and images for: Evaluation of combination protocols of the chemotherapeutic agent FX-9 with azacitidine, dichloroacetic acid, doxorubicin or carboplatin on prostate carcinoma cell lines
Source: PLoS One. 2021 Aug 25;16(8):e0256468. doi: 10.1371/journal.pone.0256468 (PMC8386839; doi:10.1371/journal.pone.0256468)

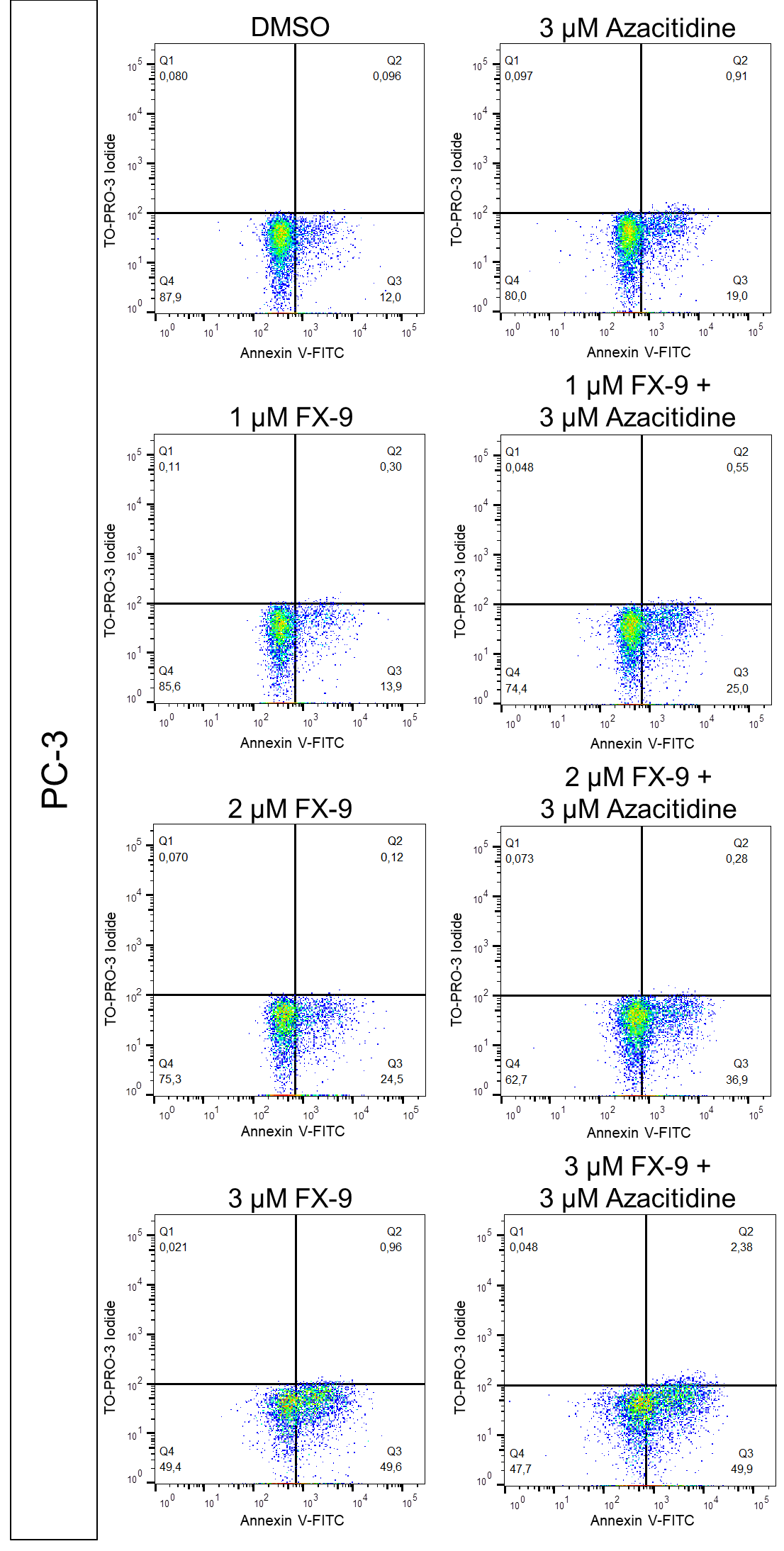

Supplement: S1 Fig — PC-3 was stained with Annexin V-FITC and TO-PRO-3 iodide after exposure to FX-9 and azacitidine. Cells in area Q4 were counted as vital, cells in Q3 as apoptotic, and cells in Q1 and Q2 as necrotic. (TIF) [file pone.0256468.s001.tif]

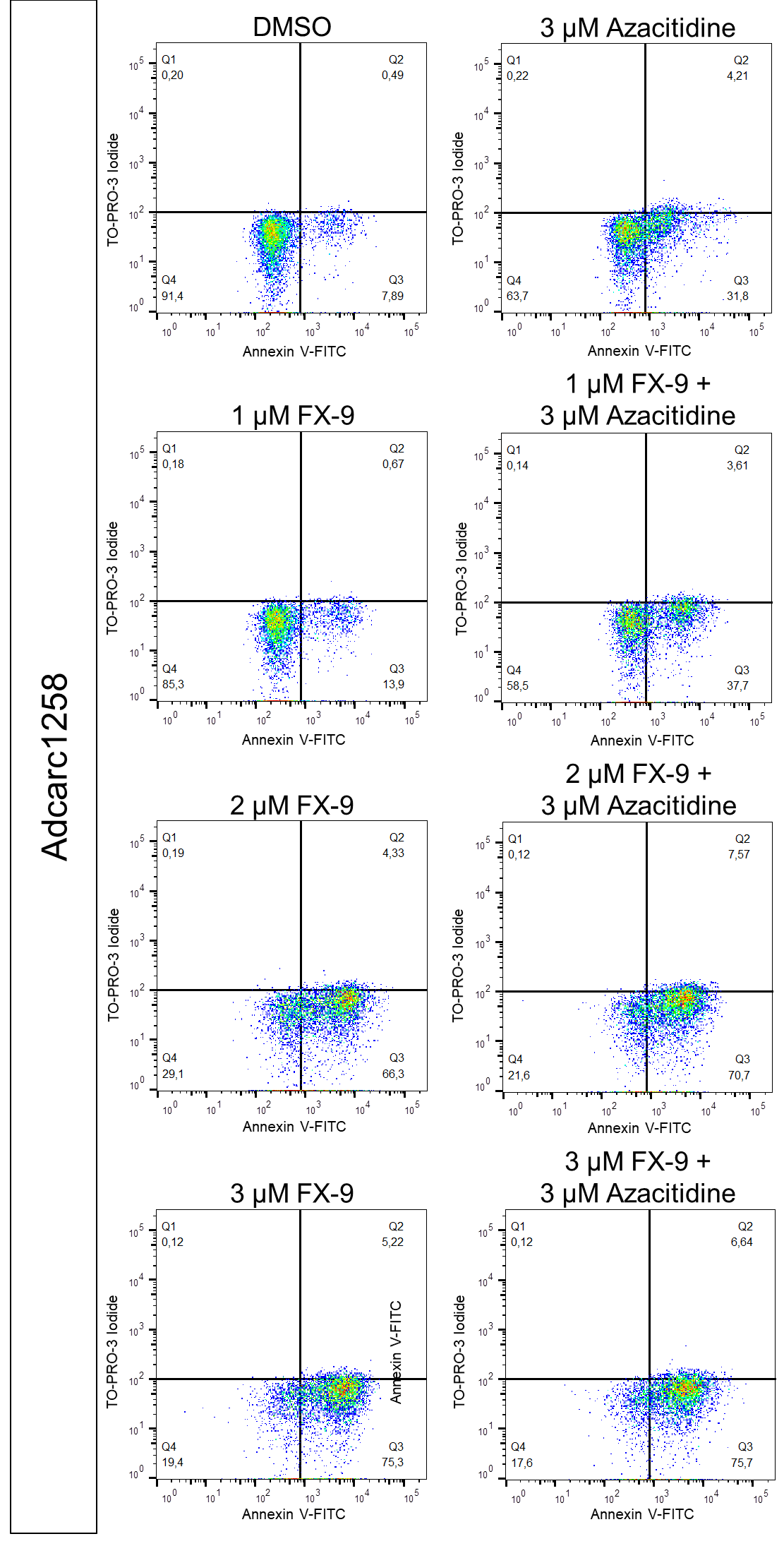

Supplement: S2 Fig — Adcarc1258 was stained with Annexin V-FITC and TO-PRO-3 iodide after exposure to FX-9 and azacitidine. Cells in area Q4 were counted as vital, cells in Q3 as apoptotic, and cells in Q1 and Q2 as necrotic. (TIF) [file pone.0256468.s002.tif]

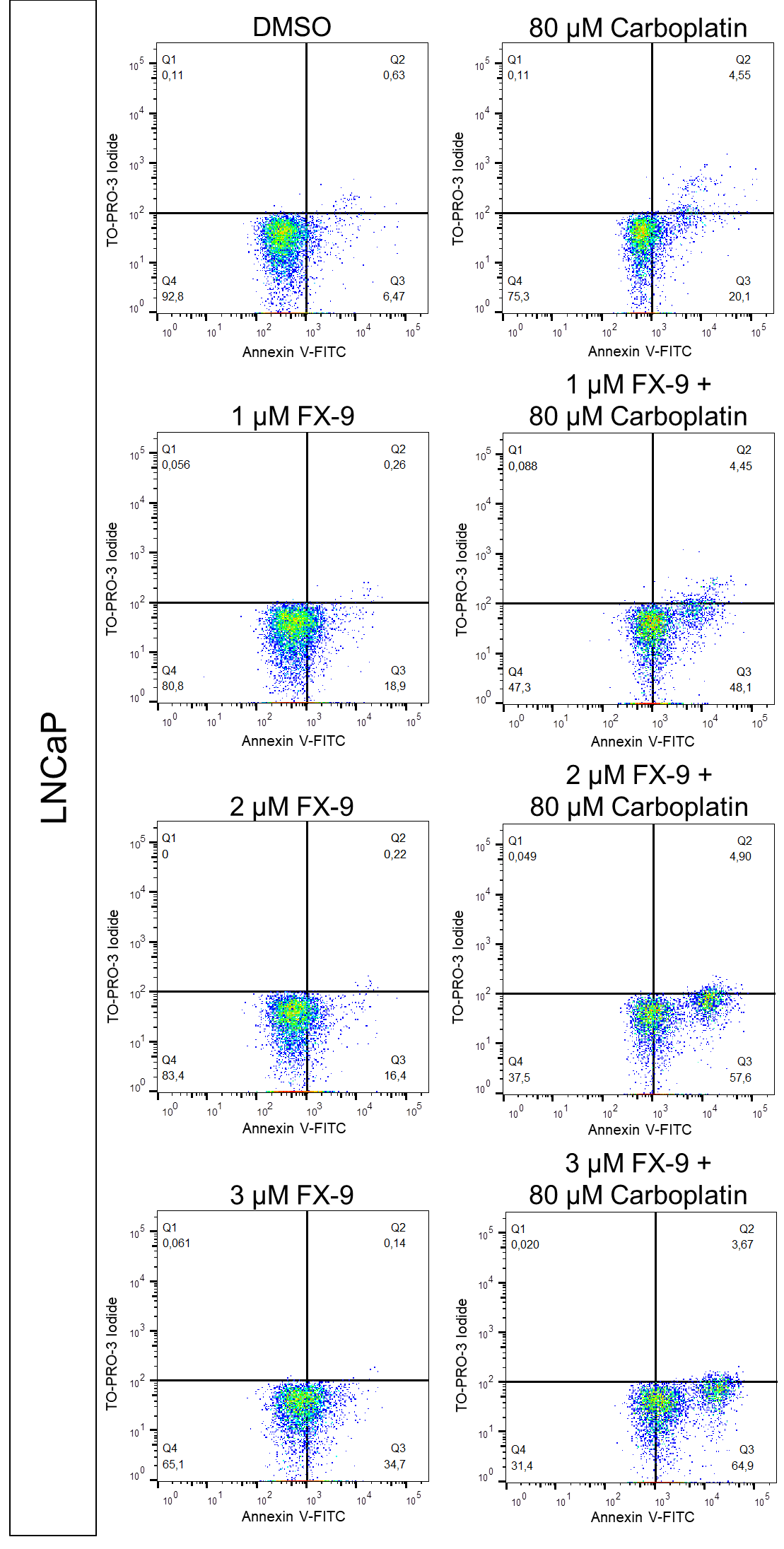

Supplement: S3 Fig — LNCaP was stained with Annexin V-FITC and TO-PRO-3 iodide after exposure to FX-9 and carboplatin. Cells in area Q4 were counted as vital, cells in Q3 as apoptotic, and cells in Q1 and Q2 as necrotic. (TIF) [file pone.0256468.s003.tif]

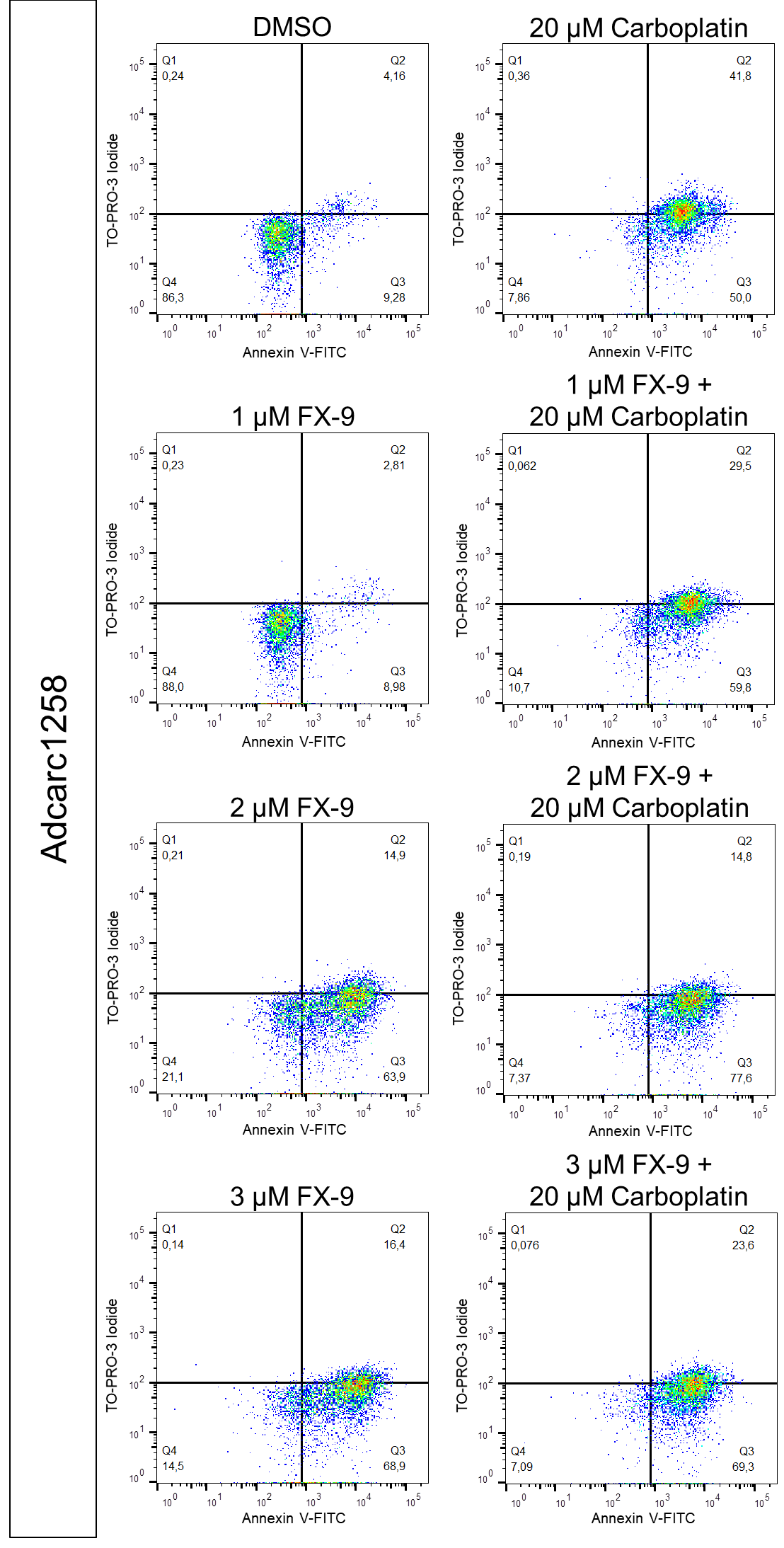

Supplement: S4 Fig — Adcarc1258 was stained with Annexin V-FITC and TO-PRO-3 iodide after exposure to FX-9 and carboplatin. Cells in area Q4 were counted as vital, cells in Q3 as apoptotic, and cells in Q1 and Q2 as necrotic. (TIF) [file pone.0256468.s004.tif]
