# Supplementary material for: Evaluation of combination protocols of the chemotherapeutic agent FX-9 with azacitidine, dichloroacetic acid, doxorubicin or carboplatin on prostate carcinoma cell lines
Source: PLoS One. 2021 Aug 25;16(8):e0256468. doi: 10.1371/journal.pone.0256468 (PMC8386839; doi:10.1371/journal.pone.0256468)
Supplement: S2 Table — (DOCX) [file pone.0256468.s006.docx]

**S2 Table. Effect of combinations on cell count.**

|  | 1 µM FX-9 | 2 µM FX-9 | 3 µM FX-9 |
| --- | --- | --- | --- |
| azacitidine | x | x | x |
| carboplatin | x | x | x |

Significant decrease in cell viability by combination of 1-3 µM FX-9 with azacitidine or carboplatin compared to DMSO-control in PC-3, LNCaP and Adcarc1258. Significance was calculated by Dunnett’s t-test. x:p<0.05
