# Supplementary material for: Evaluation of combination protocols of the chemotherapeutic agent FX-9 with azacitidine, dichloroacetic acid, doxorubicin or carboplatin on prostate carcinoma cell lines
Source: PLoS One. 2021 Aug 25;16(8):e0256468. doi: 10.1371/journal.pone.0256468 (PMC8386839; doi:10.1371/journal.pone.0256468)
Supplement: S3 Table — (DOCX) [file pone.0256468.s007.docx]

**S3 Table. Bliss values of the cell count.**

| **A** azacitidine | Bliss values | | |
| --- | --- | --- | --- |
|  | 1 µM FX-9 | 2 µM FX-9 | 3 µM FX-9 |
| Adcarc1258 | 0 | -0.1 | -0.1 |
| PC-3 | -0.1 | 0 | -0.1 |

| **B** carboplatin | Bliss values | | |
| --- | --- | --- | --- |
|  | 1 µM FX-9 | 2 µM FX-9 | 3 µM FX-9 |
| Adcarc1258 | 0 | -0.1 | -0.1 |
| LNCaP | 0 | 0 | -0.1 |

Bliss values calculated from cell count after exposure to the combination of FX-9 with **A** azacitidine and **B** carboplatin.
