# Supplementary material for: Evaluation of combination protocols of the chemotherapeutic agent FX-9 with azacitidine, dichloroacetic acid, doxorubicin or carboplatin on prostate carcinoma cell lines
Source: PLoS One. 2021 Aug 25;16(8):e0256468. doi: 10.1371/journal.pone.0256468 (PMC8386839; doi:10.1371/journal.pone.0256468)
Supplement: S4 Table — (DOCX) [file pone.0256468.s008.docx]

**S4 Table. Effect of combinations on cell fractions.**

|  | vital cells | | | apoptotic cells | | | necrotic cells | | |
| --- | --- | --- | --- | --- | --- | --- | --- | --- | --- |
|  | 1 µM  FX-9 | 2 µM  FX-9 | 3 µM  FX-9 | 1 µM  FX-9 | 2 µM  FX-9 | 3 µM  FX-9 | 1 µM  FX-9 | 2 µM  FX-9 | 3 µM  FX-9 |
| azacitidine | x | x | x | x | x | x | (x) | (x) | x |
| carboplatin | x | x | x | x | x | x | x | [x] | [x] |

Significant decrease in cell viability by combination of 1-3 µM FX-9 with azacitidine or carboplatin compared to DMSO-control in PC-3, LNCaP and Adcarc1258. Significance was calculated by Dunnett’s t-test. x:p<0.05; (x):p<0.05 PC-3 excluded; [x]:p<0.05 Adcarc1258 excluded
