# Supplementary material for: Evaluation of combination protocols of the chemotherapeutic agent FX-9 with azacitidine, dichloroacetic acid, doxorubicin or carboplatin on prostate carcinoma cell lines
Source: PLoS One. 2021 Aug 25;16(8):e0256468. doi: 10.1371/journal.pone.0256468 (PMC8386839; doi:10.1371/journal.pone.0256468)
Supplement: S5 Table — (DOCX) [file pone.0256468.s009.docx]

**S5 Table. Bliss values of the analysis of apoptosis.**

| **A** azacitidine |  | Bliss values | | |
| --- | --- | --- | --- | --- |
|  |  | vital cells | apoptotic cells | necrotic cells |
| PC-3 | 1 µM FX-9 | -0.1 | 0.2 | 0 |
|  | 2 µM FX-9 | 0 | 0.3 | 0 |
|  | 3 µM FX-9 | -0.1 | 0.4 | 0 |
| Adcarc1258 | 1 µM FX-9 | 0 | 0.3 | 0 |
|  | 2 µM FX-9 | 0 | 0.5 | 0.1 |
|  | 3 µM FX-9 | -0.1 | 0.5 | 0.1 |

| **B** carboplatin |  | Bliss values | | |
| --- | --- | --- | --- | --- |
|  |  | vital cells | apoptotic cells | necrotic cells |
| LNCaP | 1 µM FX-9 | 0.1 | 0.4 | 0 |
|  | 2 µM FX-9 | 0.1 | 0.4 | 0 |
|  | 3 µM FX-9 | 0.1 | 0.5 | 0 |
| Adcarc1258 | 1 µM FX-9 | 0 | 0.6 | 0.3 |
|  | 2 µM FX-9 | -0.1 | 0.4 | 0.1 |
|  | 3 µM FX-9 | -0.1 | 0.4 | 0.1 |

Bliss values calculated from fraction of vital, apoptotic and necrotic cells after exposure to the combination of FX-9 with **A** azacitidine on PC-3, Adcarc1258, and **B** carboplatin on LNCaP and Adcarc1258.
